# Legal frameworks and practical challenges: a review of the enduring failure to prevent family separations in armed conflicts

**DOI:** 10.3389/fpsyg.2025.1629843

**Published:** 2025-11-05

**Authors:** Neil Boothby

**Affiliations:** Global Center for the Development of the Whole Child, University of Notre Dame, Notre Dame, IN, United States

**Keywords:** separated children, unaccompanied children, armed conflict, humanitarian response, child rights, international humanitarian law

## Abstract

The separation of families during armed conflicts remains a persistent tragedy, inflicting suffering on individuals and tearing apart the social fabric of communities. Despite the existence of international laws and conventions designed to protect families and ensure their reunification, the international community continues to fall short in preventing these separations. This review seeks to analyze the multifaceted reasons behind this ongoing challenge by examining the relevant international legal frameworks and the limitations and practical challenges faced by local, national, and international actors. It further seeks to explore the underlying causes of separation, and unintended consequences of humanitarian responses, drawing on lessons learned from past and present conflicts, in an effort to illuminate potential pathways to more effective actions.

## Introduction

UNICEF estimates that over 473 million children—nearly one in five globally—are currently living in or fleeing conflict zones (Østby and Siri Rustad, 2024; UNICEF USA, n.d.). Within this war-affected child population are those who have become separated from their families. The phenomenon of children being separated during wartime is not new (Ressler, 1988). Throughout history, armed conflicts have forcibly displaced populations, leading to family fragmentation. However, the scale and nature of family separation have evolved with modern warfare, a crisis underscored by estimates that hundreds of thousands of children are currently separated from their families globally due to conflict, contributing to what some term a “global crisis” (Save the Children, 2023; PBS News, 2020).

Children separated from their families in armed conflict face an array of specific risks, exacerbating the already dire circumstances of war. These children, defined as those under 18 years of age who are apart from their parents or primary caregivers, are plunged into a state of extreme vulnerability, making them susceptible to a cascade of threats that can have devastating and long-lasting consequences (International Committee of the Red Cross, 2004). The absence of parental protection immediately elevates a child’s risk profile. Separated children are at a significantly higher risk of violence, exploitation, abuse, and recruitment into armed forces or groups. This vulnerability stems from various factors. Families fleeing violence can become separated during chaotic movements, bombings, or attacks, leaving children disoriented and alone. This disarray creates opportunities for predators and makes children easy targets. Conflict often results in the loss of adult family members, leaving children orphaned or without care, effectively removing their primary source of protection. Both boys and girls are actively recruited or abducted by armed forces and groups, often far from their homes. They may be forced into roles such as combatants, porters, spies, cooks, or subjected to sexual exploitation. Children associated with armed groups, or even those merely perceived to be, may be detained, leading to separation from their families and exposure to further risks within detention facilities (Wessells, 2016).

The psychological effects of war and family separation were recognized early in the field of child psychology. The classic work of Burlingham and Freud (1944), based on observations of children in British nurseries during World War II, was foundational in demonstrating the profound psychological effects of war and family separation on children, even in the absence of direct physical injury. Their research highlighted that the emotional turmoil of separation and the disruption of attachment bonds could be as damaging as, if not more damaging than, physical harm (Burlingham and Freud, 1944). More recent systematic reviews corroborate and expand upon these findings. They provide a detailed examination of the relationship between exposure to war, forced displacement, and mental health outcomes in children, identifying key risk factors that are often exacerbated by family separation (Betancourt et al., 2010; Tol et al., 2013). They documented a range of troubling outcomes that include PTSD, depression, and behavioral problems.

For separated children, these problems are particularly acute, as the very structure of their support system—the family—is absent, leaving them to navigate war-related events largely alone (Brown et al., 2017). A systematic review that specifically synthesizes research on the psychosocial well-being of separated children highlights the negative effects of family separation on their development and mental health (Jones-Mason et al., 2019). These developmental impacts can manifest in various ways, including difficulties in forming secure attachments, impaired cognitive development due to chronic stress, and challenges in emotional regulation. The absence of consistent parental guidance and a stable home environment deprives children of crucial developmental stimuli and protective factors, essential for healthy growth and resilience.

Beyond individual psychological distress, family separation in armed conflict leads to significant social consequences, disrupting a child’s social integration, identity formation, and overall protective environment. Children separated from their families often face immense challenges in forming new social connections and integrating into communities, whether in displacement camps, host communities, or later upon return or resettlement. A review of war-affected youth noted the prevalence of social difficulties and behavioral problems among young people affected by war (Brown et al., 2017; Tol et al., 2013). For separated children, these difficulties are amplified by their isolated status, making it harder to establish trust, understand social norms in new contexts, and access social support networks. The absence of family structures also profoundly affects a child’s sense of identity and belonging. Children who are separated from their cultural roots and family narratives can struggle with identity formation, leading to feelings of alienation and confusion. Moreover, the lack of family protection significantly increases their vulnerability to various forms of exploitation, abuse, and neglect. A meta-analysis emphasizes the need for coordinated and evidence-based policy and practice for protecting children outside of family care (Boothby et al., 2012; Boothby, 2008). These finding highlight the systemic recognition that separated children are in an exceptionally precarious position, requiring specialized human rights and programmatic interventions to mitigate the risks they face.

## Method: scoping review on prevention of family separation in armed conflict

This review seeks to analyze the multifaceted reasons behind the enduring challenge to prevent child-family separations in armed conflict. This specific focus on the prevention of family separation during armed conflict was identified as a key concern in consultation with the Humanitarian Development Partnership’s (HDPI) working group on child protection (HDPI, n.d.). HDPI’s child protection working group serves as an informal advisory group to humanitarian practitioners, policy makers, and scholars concerned with child protection responses in situations of armed conflict and mass population movements. It’s membership includes 11 experts—former child protection leaders in UNICEF, UNHCR, United Nations Development Program, Save the Children, International Rescue Committee, Child Fund, and USAID—with significant practice and policy experience with separated and unaccompanied children in armed conflict. Its non-affiliation status allows the group to render impartial advice—in this case—to help frame the study’s focus. An initial consultation reviewed a range of critical issues including interim care, family tracing and reunification, and the fate of child protection in general given recent bilateral funding constraints. While these concerns were thoroughly vetted, the strongest consensus between the author and this informal advisory group emerged around questions involving family unity and the inability to prevent family separation during armed conflicts.

The PCC framework (population concept, and context) is recommended as a guide to construct clear and meaningful objectives and eligible criteria for a scoping review (Pollock et al., 2023), and was employed to frame this study. (1) *Population*: the population of concern is separated and unaccompanied children; (2) *Concept*: the purpose of the study is to identify the rights to family unity and protection guarantees for separated and unaccompanied children enshrined in international humanitarian law and human rights conventions and obstacles to enforcing these protection measures; and, (3) *Context*: the study’s focus is limited to armed conflicts mass population movements. The PCC framework was shared with the HDPI child protection working group during a second consultation to frame a discussion on key research questions. An overarching research question emerged from this second discussion: What factors contribute to the enduring challenge to effectively prevent family separation in the context of armed conflict? Sub research questions were also identified: (1) What are the relevant legal and policy frameworks intended to prevent family separation in armed conflict, and what are the reported limitations in their practical application? (2) What operational and logistical challenges do humanitarian actors face in their efforts to prevent family separation during armed conflict? And, (3) What evidence exists regarding the unintended consequences of humanitarian approaches and programs as they relate to family separation?

A scoping review method was used for this study to map the existing literature on the enduring challenge to prevent child-family separations in armed conflict. Unlike a systematic review, which answers a specific, focused question, a scoping review aims to identify and analyze the breadth of inter-related questions using both published and grey literature (Arksey and O’Malley, 2005). The literature search used specialized academic databases for social sciences and international relations. Academic databases included in the search were Social Services Abstracts, PubMed/MEDLINE, and Web of Science. Google Scholar was also used to cast a wide net of scholarly literature and non-peer reviewed content. Given the nature of this topic, a grey literature search figured prominently in this scoping review and was extended to documents and reports from organizations “working on the ground,” including United Nations, International Committee of the Red Cross, international non-governmental organizations, practitioner research network, and interagency working groups.

The search terms were based on a predefined set of keywords and themes. Initial search terms included: “separated children armed conflict,” “unaccompanied children armed conflict,” and “children refugees armed conflict.” To refine the scope and focus on specific aspects of the problem, these terms were further developed into key thematic clusters. The refined search terms focused on: (1) *Prevention and intervention*: “prevention of family separation,” “family tracing and reunification,” and “unintended consequences intervention.” (2) *Child rights and legal frameworks*: “separated children humanitarian law” and “separated children human rights”.

*Studies were included if they*: (1) Focus on the separation of families (broadly defined to include nuclear, extended, and chosen families where applicable) as a direct or indirect consequence of armed conflict. (2) Examine the factors that contribute to family separation, including armed conflict, forced displacement, conflict-related deaths, recruitment of child soldiers, arbitrary detention, and lack of access to humanitarian aid. (3) Evaluate the effectiveness of the application of international humanitarian law or human rights, care and protection interventions, and/or policies aimed at preventing family separation or facilitating reunification.

*Studies were excluded if they*: (1) Focus on the psychological or social consequences of family separation, without addressing the factors leading to the separation itself. (2) Deal with family separation in contexts other than armed conflict (e.g., economic migration, natural disasters, unless these are intertwined with conflict). (3) Are not available in English.^1^ (4) Are primarily journalistic accounts or personal narratives, unless they provide data or analysis relevant to the research questions.

The initial search yielded 137 sources. An AI tool (Notebook LM) was employed to produce summaries of each source, which were spot-checked for accuracy by the author. Based on this secondary review, 34 sources that did not clearly fit inclusion criteria were eliminated, resulting in a final total of 105 sources included in this article.

## The foundation of international law

The right to family unity is a universally recognized human right, enshrined in a variety of international legal instruments Key among these is the Universal Declaration of Human Rights of 1948, which, along with the Office of the High Commissioner for Human Rights (1966), affirms that “the family is the natural and fundamental group unit of society and is entitled to protection by society and the State” (UN, n.d.; Office of the High Commissioner for Human Rights, 1989; Office of the High Commissioner for Human Rights, 1966). This principle is further reinforced by the Convention on the Rights of the Child (CRC), a pivotal document that mandates states to facilitate family reunification, particularly for migrant children (OHCHR, 1989; UNICEF, 1989).

Beyond human rights law, international humanitarian law (IHL) also establishes measures to prevent family dispersion and facilitate reunification in times of conflict (International Organization for Migration, 2021). The ICRC’s customary IHL study explicitly states that “family life must be respected as far as possible” (International Committee of the Red Cross, n.d.2). The Central Tracing Agency of the ICRC, along with the broader Red Cross and Red Crescent movement, has a specific mandate to assist in searching for family members and enabling their reunification (International Committee of the Red Cross, n.d.1; International Committee of the Red Cross, n.d.6). This legal framework mandates that the protection of families in conflict is not an optional goal but a core obligation under international law (International Committee of the Red Cross, n.d.4; International Committee of the Red Cross, n.d.5) (see Table 1).

**Table 1:** Summary of the key international legal instruments relevant to family unity and family reunification.

Instrument	Article/Rule	Key provision	Supporting sources
Universal Declaration of Human Rights	Article 16 (3)	“The family is the natural and fundamental group unit of society and is entitled to protection by society and the state”	UN (n.d.)
International Covenant on Civil and Political Rights	Article 23 (1)	“The family is the natural and fundamental group unit of society and is entitled to protection by society and the state”	Office of the High Commissioner for Human Rights (1966)
Convention on the Rights of the Child	Articles 9, 10, 22	States must ensure that children shall not be separated from their parents, respect the right to family reunification, and provide appropriate protection for refugee children	Office of the High Commissioner for Human Rights (1989) and UNICEF (1989)
Customary International Humanitarian Law	Rule 105, 131	“Family life must be respected as far as possible.” All possible measures must be taken so that “members of the same family are not separated”	International Committee of the Red Cross (n.d.1) and International Committee of the Red Cross (n.d.2)

### Humanitarian guiding principles and actions

Building on the legal foundation, humanitarian organizations have developed practical frameworks to guide their interventions. The Inter-Agency Guiding Principles on Unaccompanied and Separated Children, a consensus document prepared by leading humanitarian bodies including the ICRC and UNICEF, serves as a comprehensive roadmap for action in emergencies (International Committee of the Red Cross, 2004). These principles cover all stages of a crisis, from preventing separation to tracing, reunification, and long-term care. More recently, NGO working groups have focused on prevention of child recruitment and use of children by armed forces and armed groups (The Alliance for Child Protection in Humanitarian Action, n.d.; Interaction, 2025).

In practice, humanitarian agencies like UNICEF focus their efforts on strengthening child protection systems to prevent and respond to abuse and exploitation (UNICEF USA, n.d.). This involves supporting families, equipping community-based groups, and providing direct emergency relief, such as medical care, safe water, and psychosocial support. A pivotal aspect of this work is the direct care for and reunification of children who have been separated from their families (International Committee of the Red Cross, 2004; International Organization for Migration, 2021). The process of family tracing, identification, and reunification is a key component of the guiding principles, with an emphasis on acting quickly and safely to ensure the best interests of the child (Global Protection Cluster, n.d.). This framework moves beyond abstract legal rights to a concrete set of actions designed to protect and care for vulnerable children in humanitarian crises (UN Research Guides, 2025).

## The anatomy of separation: understanding the diverse causes in conflict zones

Despite a comprehensive legal framework and practical guidelines, the continued challenge to effectively prevent family separations in armed conflicts suggests a significant disconnect between the existence of legal obligations and the recognition of those obligations on the ground. Indeed, the number of separated children in conflict zones continues to grow, with estimates upwards to 150,000 worldwide (War Child, 2024). To be sure, the tragedy of family separation in conflict zones is not a monolithic phenomenon; rather, it is the result of a complex interplay of systemic failures, strategic actions, and chaotic circumstances. This section delves into the specific, identifiable mechanisms that lead to children becoming separated from their families. Based on the findings and analyses of this scoping review, the causes outlined herein have been identified as the main, recurring factors driving family separation and the subsequent endangerment of children in contexts of armed conflict and violence.

### Mass displacement

Rapid, violent displacement can shatter family units and stands as a primary driver of child-family separations (Better Care Network, 2018; Alexander et al., 2010). In the chaos of fleeing conflict zones, families can easily become separated, whether during the initial flight, in overcrowded shelters, or while attempting to cross borders in search of safety (Civilians in Conflict, 2023). Displacement not only separates families physically but also disrupts their established support networks, leaving individuals, particularly children, more vulnerable to a range of risks.

The Rohingya refugee crisis is a case in point. In August 2017, a mass exodus of the Rohingya people began from Myanmar’s Rakhine State into Bangladesh, triggered by a brutal military crackdown. More than 700,000 Rohingya, over half of whom were children, fled their homes in a matter of weeks (UNHCR, 2017). The journey was perilous, with families often fleeing on foot or by boat under a hail of violence and persecution. In the panic and disorder, countless children were separated from their parents or caregivers (Save the Children, 2017). The violence was so intense that some children arrived in Bangladesh having witnessed the deaths of their parents or other family members. The sheer volume of people crossing the border made it impossible to keep track of everyone. Humanitarian organizations established special centers and tracing programs to help identify and reunite unaccompanied and separated children. However, the sheer scale of the displacement, coupled with the horrific experiences of both children and parents, made these efforts incredibly difficult. Many children, having been orphaned or separated, were left to care for younger siblings, forced to become heads of households overnight (UNICEF, 2022).

### Mass destruction

The humanitarian crisis in Gaza has resulted in a devastating number of separated infants and children, a situation made more complex by the extreme conditions of the conflict (UN, 2025, International Rescue Committee, 2024a). While precise numbers are difficult to verify due to the ongoing hostilities and displacement, the UN has estimated at least 17,000 children have been separated or unaccompanied (UNICEF USA, 2025). This staggering number is a direct result of relentless military operations, mass displacement, and the collapse of civil and family structures. Many children have lost their families entirely, with some being found alone in hospitals or amid rubble, unable to even recall their names (International Rescue Committee, 2024a). Mass destruction has forced families to flee their homes, multiple times, often under fire (UN, 2025). In the chaos of these evacuations, children have been separated from their parents or caregivers. The constant movement of the population, with families relocating from northern to southern Gaza and back, makes tracing efforts nearly impossible (International Rescue Committee, 2024b). The high number of civilian casualties means that many children have been orphaned. Others are separated from their parents who are injured and receiving medical care, or who have been detained. The absence of a functioning, centralized system for tracking and registering unaccompanied children is a major barrier. Humanitarian workers and community members are often working in a vacuum, with disrupted communication and no official records to assist them (UN, 2024).

### Chronicity

The chronic nature of numerous armed conflicts creates a humanitarian crisis where family separation is a widespread and long-term issue, as is the case in South Sudan (McCauley, 2024). Unlike a short-term disaster where families might be displaced for a few weeks or months, the ongoing conflict since 2013 has meant that children and parents can be separated for years, sometimes even a decade, making the process of family tracing and reunification (FTR) a complex and persistent challenge (Sandbu-Ryena, 2019). South Sudan has one of the highest rates of internally displaced people in the world, with millions of people having fled their homes, often multiple times (UNHCR, 2025). In the chaos of these repeated evacuations, families are torn apart. Children have been separated from their parents while fleeing an armed attack or while seeking food and shelter. Many end-up in different, isolated refugee camps or across international borders. The length of the conflict also means that the children who were separated years ago have now grown up, making physical identification and memory-based tracing efforts more difficult (UN, 2017). Furthermore, a new generation of separated children is continually being created as the violence flares up in different regions.

The chronicity of the conflict means that FTR cannot be a short-term emergency response. It requires a sustained, long-term commitment from humanitarian organizations. Agencies like UNICEF, Save the Children, and the International Committee of the Red Cross (ICRC) have established permanent programs in South Sudan with dedicated case workers who build trust with local communities and meticulously maintain a national database of separated children (Sandbu-Ryena, 2019). This long-term approach has yielded some successes. Since the outbreak of the war, thousands of children have been successfully reunited with their families, sometimes years after they were separated (Ferguson, 2018). The case of South Sudan demonstrates that in a protracted crisis, family reunification is not just a quick rescue mission, but a continuous, essential effort to restore the fundamental right of a child to live with their family.

### Deliberate targeting families

A tactic of war that specifically targets families is known as “kinocide.” This strategy aims to terrorize the population and destroy the social fabric of a community by intentionally harming, abducting, or killing family members of the enemy. The goal is to break the will of the opposition and create a climate of fear and submission. The Lord’s Resistance Army (LRA), for example, systematically and deliberately targeted families as a core tactic of its campaign of terror in Northern Uganda (Office of the Special Representative of the Secretary-General for Children in Armed Conflict, n.d.). The LRA’s strategy was not just to kill or abduct, but to psychologically and socially annihilate a community’s ability to resist by destroying the family unit itself. The Lord’s Resistance Army (LRA) is also notorious for its mass abductions of children from Northern Uganda and surrounding regions (Kelly et al., 2016). The LRA’s recruitment was almost entirely based on forced conscription. They would raid villages, schools, and homes, taking children and forcing them into their ranks as combatants, porters, or “wives” for commanders. The LRA’s indoctrination methods included stripping children of their former identities, given new names and forced to adopt new “family” structures within the LRA, with senior fighters acting as “fathers” (Kelly et al., 2016). These new bonds were used to replace their original family connections. The group actively prevented any contact with their former communities. For girls, the forced “marriages” and subsequent pregnancies resulted in them returning with children of their own, facing social stigma and rejection from their families and communities who saw them as being associated with the enemy. This often led to a second separation—from their children—as some communities or family members would only accept them back if they abandoned their LRA-born children.

### Forced recruitment

As highlighted with the LRA example, forced recruitment into armed forces or groups is a significant cause of family separation (International Committee of the Red Cross, 2013). Children are often targeted for recruitment, either through abduction, coercion, or manipulation, tearing them away from their families and communities (Internal Displacement Monitoring Centre, n.d.). This practice not only separates children from their loved ones but also exposes them to extreme violence, exploitation, and abuse. Many children are used by warring parties in areas that are inaccessible to United Nations officials and other observers, making it impossible to document cases, rendering the existing monitoring mechanism ineffective. For example, estimated of the number of child soldiers under the age of 18 during the Sierra Leone Civil War (1991–2002), range from 10,000 verified UN cases to over 100,000 estimated through population-based methodologies. Regardless of scale, the Revolutionary United Front (RUF) and other armed groups did systematically abducted thousands of children (Betancourt et al., 2010). The recruitment process was designed to traumatize and break the familial bonds of the victims. The forced separation was a traumatic and lasting experience for both the abducted children and their families (Schomerus, 2007; Human Rights Watch, 2000). Many parents and relatives never knew the fate of their children, living with years of uncertainty. The RUF would move abductees far from their homes to disorient them and make escape difficult. Even after the conflict ended, the process of reunification was challenging. Some children, having forgotten their old names and family structures, could not be traced. Others faced stigma and rejection from their communities and families, who struggled to accept them back after the atrocities they were forced to commit (Betancourt et al., 2010; Schomerus, 2007).

### Forced transfer

Since Russia’s full-scale invasion of Ukraine in 2022, there have been widespread reports of the illegal transportation of Ukrainian children to Russia and Russian-occupied territories (Office of the High Commissioner for Human Rights, 2025; Council of Europe, 2025). This has been documented by multiple international organizations and governments, and is a key focus of legal and diplomatic efforts. The number of children forcibly transferred is difficult to verify, with estimates ranging widely. The Ukrainian government has confirmed nearly 20,000 cases, while other sources like the Yale Humanitarian Research Lab place the figure closer to 35,000 (Yale School of Medicine, 2025). Russia’s Commissioner for Children’s Rights has claimed that over 700,000 Ukrainian children have been “relocated” (Ödebrink, 2025). The transfers often occur under the guise of “humanitarian” or “recreational” programs, such as summer camps, medical care, or evacuation from war zones. Russian authorities also target vulnerable children, including orphans, those with disabilities, or children from low-income families (European Council, 2025). There are also documented cases of children being separated from their parents, and sometimes even their parents being killed, with the children subsequently being taken to Russia. Once in Russia, Ukrainian children are subjected to a systematic program of “re-education” and Russification aimed at erasing their Ukrainian identity (Erlewein et al., 2024; Mentzelopoulou, 2025). This includes cultural erasure (must speak Russian), militarization (military camps and youth armies), and forced adoption and citizenship (recent Russian legislation streamlines the process). The European Court of Human Rights has found Russia responsible for “systematic and regulatory” violations of human rights, including the unlawful transfer of children,” and the European Union has condemned Russia’s actions and imposed sanctions on individuals and organizations involved in the illegal transfers (Council of Europe, 2025).

### Loss of identify documents

The loss or destruction of identification documentation during armed conflict presents a major obstacle to family tracing and reunification (Hague Conference on Private International Law, 2020). Without proper documentation, it becomes exceedingly difficult to establish identity and prove family relationships, especially when attempting to reunite families across international borders (American Academy of Child and Adolescent Psychiatry, 2023). Without official proof of identity, nationality, or family ties, displaced individuals, particularly children, can face bureaucratic and legal hurdles that prevent them from reconnecting with loved ones.

The Bosnian War (1992–1995) led to mass displacement, disappearances, and the systematic destruction of civil records and personal documents. Many people were killed or went missing, and their remains were buried in unmarked mass graves (Williams and Crews, 2023). The lack of identification on the bodies and the destruction of records presented an immense challenge for tracing services. The International Commission on Missing Persons (ICMP) was established to help identify the missing (International Commission on Missing Person, 2023a, 2023b). Because physical documents were often destroyed or unobtainable, the ICMP pioneered the use of DNA-based identification to match human remains with living relatives (Barnert et al., 2022). This scientific approach became a vital tool for proving the identities of the deceased and providing closure to families. However, even with this advanced method, the process was complicated and lengthy, requiring the voluntary participation of family members to provide genetic samples. While not a direct reunification in the traditional sense, this process of identification provided a form of “reunification” by confirming the fate of missing family members, which was an essential step for survivors to move forward.

The more recent Syrian civil war has caused one of the largest displacement crises in modern history, with millions of people fleeing their homes. In the chaos of flight, many refugees lost or had their identification documents, such as national ID cards, passports, and family booklets, destroyed (Bunn et al., 2023). These documents are vital for proving legal identity and family relationships. For Syrian refugees, the absence of a “family booklet,” a key civil record, has become a major challenge. This document is required to prove a person’s family lineage and is necessary for registering marriages, births, and deaths. Families who were separated and sought asylum in different countries often could not prove their relationship to one another without these booklets (McNatt and Boothby, 2018). This made it difficult, if not impossible, for children to be reunited with their parents or for spouses to join their partners through legal family reunification processes. This documentation gap has also created a risk of statelessness for children born in displacement, as they cannot be registered and issued documents without their parents’ proper identification, which in turn leads to a host of other protection issues (Bunn et al., 2023).

Examining specific instances of armed conflict provides valuable insights into the patterns of family separation and the effectiveness of the international community’s response. Collectively, these examples illustrate some of the recurring challenges associated with family separation in armed conflicts, including the massive scale of displacement, the devastating impact of forced recruitment and abduction, and the significant difficulties encountered in facilitating in-country and cross-border family reunification. The following table summarizes some of the main causes of child-family separation during armed conflict (see Table 2).

**Table 2:** Causes of child-family separation in armed conflict.

Category of cause	Specific causes of child-family separation
Direct conflict-related events	
Mass/Chaotic displacement	Children becoming separated while fleeing attacks, during initial flight, in overcrowded shelters, or while crossing borders in search of safety
Mass destruction & violence	Separation resulting from relentless military operations, bombings, or attacks Children being separated in the chaos and violence of a humanitarian emergency (sudden danger)
Death or incapacity of parents/caregivers	Conflict resulting in the loss of adult family members, leaving children orphaned Parents dying or becoming too weak/injured to continue their journey, leaving children behind, or being detained
Chronicity	Protracted conflicts creating long-term separation (sometimes a decade), with repeated evacuations and a new generation of separated children continually being created
Deliberate/Coercive actions by armed actors	
Deliberate targeting of families (kinocide)	A tactic of war aimed at terrorizing the population by intentionally harming, abducting, or killing family members of the opposition to destroy the social fabric
Forced recruitment	The abduction, coercion, or manipulation of children into armed forces or groups (e.g., as combatants, spies, porters), which breaks familial bonds
Forced transfer	A party to a conflict illegally transporting or abducting children to another country or occupied territory, often under the guise of “humanitarian” or “recreational” programs
Exploitation and detention	Parties involved in the conflict forcibly pulling children away from their families for exploitation, or children/parents being detained
Family/Parental actions (protective or desperate)	
Seeking safety	Parents choosing to send their children to a safer location or neighboring country in hopes they will find asylum and escape danger
Abandonment	Parents, in desperate situations, leaving children with others, such as in a hospital or camp, believing the child has a better chance of survival
Economic coping strategies	Disrupted family structures forcing parents to separate from their children as a negative coping mechanism
Voluntary joining/labor	Some children voluntarily leaving their families to find work or join armed groups/forces, often to lessen the financial burden on their parents
Systemic, legal, and logistical barriers	
Loss of identity documents	The loss or destruction of identification and civil records (e.g., “family booklets”) making it difficult to establish identity and prove family relationships for reunification, potentially leading to statelessness
Separation at borders	Families becoming separated at borders during asylum processes, often due to restrictive national policies and bureaucratic hurdles
Evacuations	The rapid movement of large populations during military or humanitarian evacuations, where logistical challenges and the lack of prioritization of family unity can lead to fragmentation
Unintended consequences of humanitarian aid	Perceived unfairness in the distribution of limited resources, which can strain relationships and lead to further displacement, or assistance programs that focus exclusively on the child without supporting the extended family caregivers

## A shared responsibility: the roles and actions of governments, international organizations, and NGOs

The protection of the fundamental right to family unity in times of armed conflict is not merely a legal or ethical aspiration, but a matter of practical enforcement. Crucially, the issue does not hinge on the mere possession of rights by individuals, but rather the active recognition of these rights by the duty-bearers responsible for upholding them. International legal instruments, such as the Convention on the Rights of the Child (CRC) and the Geneva Conventions, enshrine the principle that families must be protected and kept together. However, the continued failure to prevent child-family separation demonstrates a significant disconnect between the existence of these legal obligations and their consistent application on the ground. This section briefly outlines the established roles and responsibilities of the key actors—Governments (both state and non-state), United Nations agencies, and civil society organizations—in promoting family unity and preventing separation during armed conflicts. Following this overview, the analysis turns to examining the profound failures by these same actors to fulfill their mandates, which ultimately contribute to the enduring crisis of separation.

National governments bear the primary responsibility for protecting their citizens, including preventing family separation and facilitating reunification (Professionals in Humanitarian Assistance and Protection, 2021). They have the duty to enact and enforce laws that protect family rights during armed conflict and to cooperate with international efforts in this area. However, in some instances, governments are perpetrators of family separation through policies such as forced transfers or deportations (Georgetown University Collaborative on Global Children’s Issues, 2023). This highlights the critical importance of holding states accountable to their international legal obligations (Gulati and Khoso, 2013).

UNICEF provides support to separated and unaccompanied children, works to identify and register them, and advocates for their rights (UNICEF USA, n.d.). UNICEF’s effectiveness, however, is often hampered by the security and access limitations inherent in conflict zones, as well as resource constraints. UNHCR has a specific mandate to protect refugees and promote family unity (UNHCR, 2021). UNHCR works to improve access to family reunification procedures for refugees and provides support to separated refugee families (United Nations, 2020). While UNHCR’s focus is primarily on refugees, family separation is also a critical issue for internally displaced persons who may not fall under UNHCR’s direct mandate. The Office of the Special Representative of the Secretary-General for Children and Armed Conflict (Office of the Special Representative of the Secretary-General for Children in Armed Conflict, n.d.) plays a role in advocating for the protection of children affected by armed conflict, raising awareness about their plight, and fostering international cooperation to this end (United Nations Security Council, 2009; Global Action on Aging, n.d.; Asokan, 2021). The SRSG CAAC’s work seeks to keep the issue on the international agenda and encourages states and other actors to take action (Security Council, 2024).

Non-governmental organizations (NGOs) are important partners in addressing family separation. They often operate directly on the ground, providing essential assistance, conducting family tracing activities, and offering reunification services (International Committee of the Red Cross, 2013). Among NGOs, the International Committee of the Red Cross (ICRC) has a specific mandate under international humanitarian law to work for family reunification and to trace missing persons (Global Protection Cluster, n.d.).

## The limits of reach: the influence of state sovereignty and non-interference

While the preceding section outlined the roles and responsibilities incumbent upon states under international human rights and humanitarian law, the practical effectiveness of this legal framework often confronts a powerful structural impediment: the principle of state sovereignty. This section, entitled “The Limits of Reach: The Influence of State Sovereignty and Non-Interference,” shifts focus from the ideal to the reality. It describes key failures identified in this scoping review to uphold international agreements designed to preserve family unity and protect separated children, demonstrating how the invocation of state autonomy and the principle of non-interference can undermine the very protections outlined in treaties, leaving vulnerable children and families exposed to inadequate or absent legal and humanitarian support.

### State sovereignty

One of the most significant obstacles to the international community’s efforts to prevent family separation and reunite children is the principle of state sovereignty. This principle, enshrined in the UN Charter, grants States exclusive authority over their internal affairs and their borders (Jones et al., 2017; Picard Kentz & Rowe LLP, 2024). While international law on human rights and humanitarian law obliges states to protect the family unit, states can and have used sovereignty as a pretext to limit or block these efforts (Woods, 2025).

The European migration crisis and subsequent responses by various European nations offer a clear case study of how state sovereignty has been used to limit family reunification efforts, particularly for refugee and asylum-seeking children. Beginning in 2015, a large influx of asylum seekers and migrants arrived in Europe, many of whom were children, including a significant number of unaccompanied minors. Many of these children were separated from their families while fleeing conflict or violence in countries like Syria, Afghanistan, and Iraq. Humanitarian organizations began the process of identifying and tracing these children’s family members, a task made even more challenging by the lack of documentation and the dispersed nature of the families across different countries.

Despite the international legal frameworks (the Convention on the Rights of the Child and the European Convention on Human Rights) which protect the right to family life, many European states invoked their sovereign right to control their borders and immigration policies. This led to a range of practices that directly obstructed family reunification, including:

- Restrictive legal definitions of “family”: Some states narrowed their legal definition of a “family member” eligible for reunification. This often meant only nuclear family members (parents, minor children, spouses) were eligible. This narrow definition excluded a significant number of vulnerable people, such as adult siblings, grandparents, and other dependents, who are often the sole remaining relatives of a separated child (Chech, 2017).
- Administrative and Bureaucratic Obstacles: States imposed stringent and often prohibitive bureaucratic hurdles (Nabuco, 2020). These included:

◦ Lengthy processing times: Applications for family reunification took years to process, during which time a child might turn 18 and no longer be considered a minor, making them ineligible for reunification.
◦ Burden of proof: Requiring families to provide documentation that was often lost or destroyed during conflict and displacement. In some cases, states demanded expensive DNA tests to prove family relationships.
◦ Financial requirements: Forcing the refugee or asylum seeker to demonstrate they had sufficient income and housing to support their family members, which is nearly impossible for someone who has just arrived in a new country and is not permitted to work.

- Changes in legislation: As the situation evolved, some countries enacted new laws or temporary exemptions that further restricted family reunification. For example, some states implemented a waiting period of up to 3 years before a person with “subsidiary protection” (a lower form of protection than refugee status) could even apply to reunite with their family (Chech, 2017).

This use of sovereignty effectively created a legal and administrative wall that humanitarian organizations and international bodies struggled to overcome. While humanitarian organizations could offer practical assistance—such as legal aid and help with documentation—their efforts were ultimately limited by national laws (Red Cross EU Office, 2015). The case of family reunification in Europe demonstrates a recurring theme: while states are signatories to international conventions that protect human rights, they can selectively apply or circumvent these obligations under the guise of national security, border control, or migration management. This highlights a fundamental tension between the universal principles of human rights and the powerful, enduring principle of state sovereignty.

### Humanitarian intervention using force

The concept of humanitarian intervention, which involves the use of force to protect populations from gross human rights violations, lies at the heart of the tension between state sovereignty and the international community’s responsibility to protect (Brown, 2000). While there is a growing recognition of this responsibility, particularly in cases of mass atrocities that may lead to family separation, the principle of sovereignty remains a powerful constraint on the willingness of states to intervene militarily without Security Council authorization or the consent of the affected state (Woods, 2025). Even non-military forms of intervention, such as targeted sanctions or diplomatic pressure aimed at influencing a state’s policies on family protection, can be viewed as infringements on state sovereignty and may be met with resistance by the concerned government (Jones et al., 2017). States often prioritize their sovereign rights over external scrutiny of their human rights practices, limiting the tools available to the international community for preventive action.

From the outset of the Syrian conflict, for example, the Syrian government consistently invoked the principle of state sovereignty as a way to reject any form of foreign military intervention aimed at protecting its population. The Assad regime and its allies, particularly Russia, framed any potential humanitarian intervention as an illegal violation of international law and a pretext for “regime change.” This invocation of sovereignty was particularly effective in the United Nations Security Council (UNSC), where Russia and China, as permanent members with veto power, repeatedly blocked resolutions that would have authorized a military response or even imposed significant sanctions. Their arguments rested on the premise that the conflict was an “internal matter” and that any external military action would be a violation of Syria’s sovereignty. At the same time, indiscriminate shelling, chemical attacks, and siege warfare against civilian populations resulted in the death of hundreds of thousands of people and forced millions to flee their homes, leading to one of the largest refugee crises in modern history. In the chaos, countless children were separated from their families. Parents were killed, detained, or lost track of their children in the stampedes of mass evacuations.

In the Syrian case, the principle of state sovereignty, as wielded by the Assad regime and its powerful allies, acted as a nearly insurmountable barrier. It effectively paralyzed the international community’s ability to use the tools of humanitarian intervention to protect a population from gross human rights abuses. As a result, family separation became not a temporary tragedy, but a chronic and ongoing consequence of a conflict that the international community, due to a diplomatic impasse, was powerless to stop.

### State policies opposing the international framework

A profound challenge to the international framework is the existence of state policies that are in direct opposition to its core principles. The United States’ policies of family separation and child detention at its borders are a stark example of this deliberate contradiction (Human Rights Watch et al., 2024; American Immigration Council, 2025). The analysis shows that these policies use children as “weapons of deterrence,” operating on the theory that treating children poorly will dissuade parents fleeing persecution from seeking asylum (Mousin, 2019). This approach is in direct violation of the CRC’s purpose of ensuring “special care and assistance” to children and recognizing the fundamental role of the family.

Furthermore, the practice of mass detention of children and families as a “first resort” and their accelerated removal is inconsistent with the CRC’s mandate that detention should only be a “last resort and for the shortest time possible.” By failing to ratify the CRC, the United States not only abdicates its moral leadership but also invites other nations to emulate its disregard for child and family rights, a critical function of its foreign policy and international standing. This situation indicates that the primary failure of the international framework is not a lack of legal instruments but a lack of political will on the part of States to adhere to their obligations.

## Unintended consequences of humanitarian interventions on family structures

Local, national, and international organizations play a complex and crucial role in humanitarian contexts. Their work can encompass a wide range of activities, from providing emergency relief and protection services to facilitating family tracing and reunification. However, the very nature of responding to emergencies in conflict zones means that even well-intentioned actions can sometimes lead to unintended negative consequences, including the separation of children from their families (Ressler, 1988; Better Care Network, 2018). While acknowledging the often-dedicated efforts of local, national, and international actors, it is also important to examine how humanitarian responses have inadvertently contributed to child-family separations during armed conflicts.

### Broader assistance

While humanitarian aid is designed to alleviate suffering and provide essential support, the complexities of its delivery and distribution in conflict zones can sometimes lead to unintended disruptions of family units (Brown, 2000; Hope and Home for Children, n.d.). For instance, the distribution of limited resources, if not managed equitably and sensitively, can create competition or tension within families or between extended family members who are sharing the responsibility of caring for children separated from their parents (ReliefWeb, 2025). Perceived unfairness in the allocation of food, shelter, or other necessities can strain relationships and lead to further displacement or separation as individuals seek what they believe to be more equitable access to aid elsewhere. Similarly, if certain types of aid disproportionately benefit some family members over others, it can alter traditional family dynamics and create imbalances that might ultimately contribute to instability and separation (Feinstein International Center, 2017).

While a dedicated focus on separated children is crucial, the design and implementation of assistance and protection programs must also consider the broader family context (Human Rights Watch, 2023). If support is provided exclusively to the child without adequately assessing and addressing the needs of the extended family or community members who are acting as caregivers, it could inadvertently place additional burdens on them. This can lead to situations where these caregivers, already stretched thin by the conflict, are unable to sustain their support, potentially resulting in the child being moved elsewhere, further disrupting family or kinship ties. A holistic approach that recognizes the interconnectedness of family members’ needs is essential to avoid unintended negative consequences on family structures (Boothby et al., 2012).

Following the civil war in Sierra Leone, for example, a post-conflict reintegration program aimed to help former child soldiers return to their families. The program’s main problem was its individual-focused approach, which provided support, such as psychosocial counseling and vocational training, directly to the former child soldiers but largely overlooked the needs of their families (Elkhaili and Sempijja, 2025). This caused significant conflict and strained relationships, ultimately hindering successful reintegration for many children. Reintegrating children, for instance, were given a small financial demobilization package and access to education or job training. However, their families, who had lost livelihoods and assets during the war, received no equivalent support. This disparity led to resentment, as families felt the returning child was receiving preferential treatment. Many former child soldiers, especially girls who had been subjected to sexual violence or forced “bush marriages,” returned with a deep sense of shame and stigma. The program’s focus on the individual did little to prepare the family and broader community to accept and support them. Without a family unit that could provide a buffer against community rejection, the children felt isolated and unwelcome, which sometimes drove them back to armed groups or to a life on the streets (Betancourt et al., 2010).

The case of Sierra Leone demonstrates that a successful reintegration program cannot treat a child in isolation. By neglecting the family unit, the program unintentionally created an environment of discord and instability (Zack-Williams, 2001). This often led to the failure of the reintegration process, with some children being re-recruited by armed groups or struggling with long-term mental health issues and social isolation.

### Institutional care during conflict

Supporting orphanages in conflict zones presents a significant dilemma for humanitarian organizations. This is particularly salient given the increasing evidence that favors family-based care as the optimal environment for children’s growth and development (St. Petersburg-USA Orphanage Research Team, 2009). Prioritizing institutional care can contradict the fundamental ethical principles of humanitarian action, such as “do no harm” and acting in the “best interests of the child” (UNICEF, 2021). Furthermore, international experience has shown that crisis response and the influx of international aid can have negative impacts on underfunded social service systems, potentially perpetuating a reliance on institutional models rather than fostering more sustainable, family-centered approaches (ReliefWeb, 2025).

Armed conflict destroys local economies, pushing families to make difficult decisions regarding the care of their children (Hope and Home for Children, n.d.). Loss of livelihoods, displacement, and the absence of social safety nets, can become a primary driver for placing children in orphanages (Feinstein International Center, 2017). In situations where families are struggling to access basic necessities such as food, shelter, healthcare, and education, orphanages might be perceived as the only viable option to ensure their children’s survival and well-being (Ressler, 1988; Better Care Network, 2018).

When families face direct threats from violence or bombardments, orphanages can be viewed as places of refuge that can provide a more secure environment for children compared to the dangers they face within their communities or while fleeing. However, this perception can be misleading. Research consistently shows that institutional care itself poses significant risks to children’s physical, emotional, and social well-being (Habbach, 2020). Moreover, orphanages in conflict zones are not immune to the dangers of war and can be susceptible to abusive conditions, exploitation, and even trafficking (van Dore and Nhep, 2021). Relying on orphanages as a primary safety measure can inadvertently expose children to other forms of harm and lead to separation from families who might be able to provide better protection with adequate support (Human Rights Watch, 2020).

In emergency situations, local and international actors often establish temporary shelters or registration points where separated children are brought for immediate care and safety. However, if these initial safety measures are not rapidly followed by robust efforts to trace and reunite children with their families, these temporary arrangements can inadvertently evolve into longer-term institutional placements (Physicians for Human Rights, 2020).

The humanitarian response in the aftermath of the 1994 Rwandan genocide is illuminating. In the immediate aftermath of the genocide, the international community and NGOs rushed to establish orphanages and “Children’s Centers” to provide care for the tens of thousands of children who had been separated from their families. While the intent was to provide a safe haven, the institutional model of care created serious problems (Human Rights Watch, 2003).

- *Disincentive for family reunification*: Orphanages, often well-funded by foreign donors, provided a level of food, shelter, and medical care that was often superior to what struggling Rwandan families could offer. This created a perverse incentive. In some cases, families who had initially taken in a separated child spontaneously—as per the traditional Rwandan practice of “spontaneous fostering”—were later unable to cope with the economic burden. They would then turn the child over to a well-resourced orphanage, believing it was a better solution for the child’s well-being. This effectively pulled children out of families and into institutional care (Waddington, 2020).
- *Stigma and psychological harm*: Children in institutions often experienced a sense of being “separate” from society. They were segregated from their communities and struggled to develop the social and relational skills needed for a normal life. This led to difficulties in reintegrating with families later, as the children had become institutionalized and often felt a disconnect from their cultural roots and family ties. Some children were reluctant to leave the “nicer” environment of the orphanages, further complicating reunification efforts (SOS Children’s Villages, 2025).
- *Perpetuation of institutions*: The availability of funding for orphanages led to a proliferation of these institutions, even as the government and aid agencies began to recognize that family-based care was the ideal and most sustainable solution. The sheer number of children in institutional care became a self-perpetuating problem, as agencies had to maintain these facilities and often delayed family reunification due to a fear that funding would cease once the number of “orphans” decreased.
- *Long-term consequences and costs*: Child-family separations continued for several years. By 1999, it was estimated that approximately 375,000 children had been orphaned or separated from their families. The Government of Rwanda became increasing concerned about the proliferation of orphanages as the cost of caring for a child in an orphanage was believed to be 7–10 times higher than providing support to a family to care for a child, due to overhead costs, including infrastructure, salaries for staff, and centralized food and medical supplies (Waddington, 2020). In contrast, family-based care models, such as foster care or support for extended families, require far less capital and are more sustainable. The Rwandan government realized the situation was not sustainable and directed international community to reorient their funding and programming to provide basic support like food aid, school fees, and medical assistance directly to the family (see Table 3).

**Table 3:** The role of orphanages in separation: comparison of institutional care and family-based care for children in conflict zones.

Criteria	Institutional care	Family-based care	Relevant references
Emotional well-being	Higher risk of emotional detachment, difficulties forming secure attachments	Promotes secure attachment and emotional stability	Burlingham and Freud (1944) St. Petersburg-USA Orphanage Research Team (2009) Ressler (1988)
Social development	Delayed social skills, challenges in forming healthy relationships	Fosters healthy social skills and positive peer interactions	Human Rights Watch (2020) St. Petersburg-USA Orphanage Research Team (2009) Better Care Network (2018)
Risk of abuse & neglect	Increased vulnerability to physical, emotional, and sexual abuse and neglect	Generally lower risk of abuse and neglect in well-supported systems	UNICEF (2021) Habbach (2020)
long-term outcomes	Child: Potential for long-term psychological and emotional issues, developmental delays Systems: Perpetuation of an institutional care model beyond the duration of a conflict	Child: Better long-term psychosocial outcomes, improved educational attainment Systems: support for alternative family care long-term model.	St. Petersburg-USA Orphanage Research Team (2009) Better Care Network (2018) Ressler (1988)
Family contact	Often limited or no family contact, weakening of family ties	Maintains crucial family ties and cultural connections	Ressler (1988) Habbach (2020)
Impact on identity	Challenges in forming a strong sense of identity and belonging	Supports the development of a strong sense of identity and cultural heritage	Ressler (1988) Habbach (2020) Better Care Network (2018)
Cost-effectiveness	Can be more expensive to maintain in the long term	Often more cost-effective and sustainable, especially when community resources are leveraged	Waddington (2020) Ressler (1988)

### Evacuations

The process of evacuating children from active conflict zones, while ostensibly a measure of protection to prevent physical harm, was identified in the scoping review as a critical issue in need of further analysis due to its frequently detrimental, unintended consequences. This section, explores this complex and often controversial practice. Historically, evacuations have been employed as an immediate life-saving tactic; however, a closer examination reveals that, more often than not, these efforts—particularly when children are moved without their parents or guardians—have inadvertently undermined family unity and resulted in lasting harm to the children and their families. To illustrate this challenge, this section provides a case illustration highlighting these unintended consequences and, drawing upon the evidence gathered in the scoping review, concludes by offering targeted guidance for designing evacuation processes that better adhere to and protect the principle of family unity.

Evacuations in conflict zones can take various forms, each carrying its own risks of family separation. These include military evacuations, often prioritized for strategic reasons; humanitarian evacuations, organized by aid organizations as a measure of last resort; and self-evacuations, where civilians flee independently seeking safety (ReliefWeb, 2025). Regardless of the initiator, the rapid movement of large populations in insecure and often hostile environments inherently increases the likelihood of families becoming separated. Logistical challenges, communication breakdowns, and the sheer scale of displacement can all contribute to family fragmentation. Furthermore, the different priorities and operational procedures of military and humanitarian actors might not always align with the primary need to keep families together. These threats and challenges underscore the importance of prioritizing the prevention of family separation as a central objective of all evacuation efforts (Abdul Waajid and Shrivastava, 2017) This need is further evidenced by the historical reality that many evacuated children were never returned to their biological families (Sharp, n.d.; Physicians for Human Rights, 2020; Ressler, 1988).

The Nigerian Civil War (1967–1970) serves as a poignant example of how the evacuation of children can lead to permanent family separation, with many children never being reunited with their families. During the war, the Nigerian government imposed a severe blockade on the secessionist state of Biafra. This led to widespread famine, and images of starving, skeletal Biafran children were broadcast worldwide, leading to a massive international humanitarian response (Ibhawph, 2020). Relief agencies undertook a large-scale airlift to bring in food and medicine. As part of this effort, they also evacuated thousands of severely malnourished children, primarily to neighboring African countries like Gabon and Côte d’Ivoire, for medical treatment and rehabilitation. While the evacuation was undertaken with humanitarian intentions, a significant number of these children were never returned to their families for several reasons.

Politics: the evacuation and repatriation process were deeply entangled in the politics of the war. Gabon and Côte d’Ivoire had recognized Biafra, putting them at odds with the Nigerian federal government. The Nigerian government insisted the children were “temporary evacuees” and not “refugees,” and the host countries were often reluctant to cooperate with international agencies for their return. The process was fraught with mistrust and conflicting interests.

- *Loss of identity documentation*: In the chaos of the war, accurate records of the children’s identities and their families were often not maintained. This made it incredibly difficult, if not impossible, to match a child to their family after the war.
- *Death and adoption*: While many children were successfully returned, a number of the most malnourished children died in the camps. Others were adopted by local families in their host countries. In some cases, Biafran mothers made the difficult decision to give up their children, and for some, this separation was final.
- *Child’s sense of time*: The long separation, coupled with the children’s young age and their integration into their new environments, meant that by the time a return was possible, many children had lost their memory of their birth families and their culture and formed close relationships with their “psychosocial families (see Table 4)”.

**Table 4:** Evacuations and family fragmentation: preventing family separation during humanitarian evacuations.

Stage of evacuation	Recommended actions for humanitarian organizations	Relevant references
Pre-evacuation planning	Conduct thorough family tracing risk assessments for the affected population. Establish clear and multiple communication channels with families using various accessible methods. Prioritize the evacuation of entire families together whenever logistically possible. Implement a robust and rapid registration system that carefully captures family links and relationships	UNHCR (2020) International Committee of the Red Cross (2004) International Committee of the Red Cross (n.d.1, n.d.5, n.d.6) UNHCR and UNICEF (1992)
During evacuation	Designate family-friendly waiting areas at transit points where families can stay together. Provide clear signage and guidance in multiple languages. Ensure trained personnel are available to assist families and prevent separation. Prioritize the needs of vulnerable family members, such as children, the elderly, and persons with disabilities. Maintain open communication with families throughout the evacuation process	International Committee of the Red Cross (2004) UNHCR (n.d.) UNHCR (2020) UNHCR and UNICEF (1992)
Post-evacuation	Establish clear procedures for reporting and addressing cases of separation. Prioritize family reunification as the primary goal in the immediate aftermath of evacuation, even if temporary accommodation is required. Provide safe and accessible spaces for separated children. Ensure immediate access to psychosocial support for distressed families and separated children. Continue family tracing efforts until all possible reunifications are achieved	International Committee of the Red Cross (n.d.1, n.d.2, n.d.3) UNHCR (2020) Better Care Network (2018) UNICEF (2024)

## Discussion: the enduring gap between legal obligation and practical protection

This scoping review set out to analyze the factors contributing to the enduring failure to effectively prevent family separation in armed conflicts, despite a comprehensive international legal and policy framework. The core finding is that the challenge is not one of absent law, but one of structural, political, and operational failure to consistently apply existing obligations on the ground. The disconnect between the ideal of family protection and the reality of persistent mass separation can be traced to three critical, interconnected mechanisms.

### The politicization of protection: the barrier of state sovereignty

The most significant structural impediment to preventing separation is the invocation of State Sovereignty. While International Humanitarian Law (IHL) and the Convention on the Rights of the Child (CRC) mandate the protection of the family unit, states can and do use sovereignty as a pretext to limit or block these efforts. This mechanism manifests in two critical ways:

- Paralyzing intervention: The principle of non-interference can paralyze international political bodies, as seen in the Syrian conflict, where the veto power, based on the principle of sovereignty, acted as an insurmountable barrier to intervention and protection of the civilian population. This failure allowed indiscriminate conflict tactics to lead to the separation of countless children.
- Obstructing reunification: In non-conflict receiving states, sovereignty is used to erect administrative and legal walls against reunification. Practices like imposing restrictive definitions of “family,” requiring the impossible burden of proof (documentation lost during displacement), and implementing lengthy processing times effectively sabotage the right to family life guaranteed under international conventions. This selective application of international law, as exemplified by migration policies in Europe and the US border policies, demonstrates a critical lack of political will to adhere to obligations when they clash with national migration controls.

### The paradox of aid: unintended consequences of intervention

Even well-intentioned humanitarian responses can inadvertently contribute to separation or undermine reunification efforts if not designed holistically. This review identified three major operational pitfalls:

- The perpetuation of institutional care: The immediate, crisis-driven prioritization of institutional care, such as orphanages, can contradict the fundamental ethical principles of humanitarian action, namely “do no harm” and acting in the “best interests of the child.” In places like post-genocide Rwanda, well-funded institutions created a perverse incentive, pulling children out of struggling family or spontaneous foster care arrangements and into long-term institutionalized environments that delayed or complicated reunification efforts.
- Individual-focused programming: Humanitarian aid that is too narrowly focused on the individual separated child (e.g., providing financial aid or training only to the former child soldier) can create resentment and discord within the family unit. As shown in Sierra Leone, this disparity can hinder successful reintegration, sometimes driving children back to armed groups or isolation, proving that a program cannot effectively treat a child in isolation from their social context.
- Evacuations as separation: The practice of evacuating children, while sometimes necessary for immediate life-saving purposes, has a frequent, detrimental unintended consequence: permanent family separation. When children are moved without their parents or guardians, the rapid, chaotic movement, logistical challenges, and historical difficulties in tracing and returning children often result in the undermining of family unity, which underscores the necessity of prioritizing family unity as the central objective of all evacuation efforts.

## Conclusion and recommendations

The separation of children from their families in armed conflict remains a persistent tragedy, sustained not by a void of law, but by a failure of will. The current focus on Family Tracing and Reunification (FTR), while essential, is an insufficient strategy that overwhelmingly addresses the symptoms rather than the root cause. This review concludes that the enduring crisis can only be mitigated by a fundamental shift toward prevention and political accountability.

Based on the synthesis of legal, political, and operational failures, this review offers the following recommendations for a more effective and coherent strategy:

- Strengthen accountability against state sovereignty: International bodies, including the UN Security Council, must develop mechanisms to impose sanctions or diplomatic consequences on states—both conflict actors and receiving states—that invoke sovereignty to deliberately obstruct IHL/CRC obligations regarding family protection, forced transfers, or family reunification.
- Mandate family-based care in all emergency protocols: International donors and humanitarian agencies must make it a condition of funding that responses prioritize and financially support family-based care models (kinship care, spontaneous fostering, community-based care) over institutional models, except as an absolute last resort and for the shortest duration possible. Funding must be flexible to support the economic needs of struggling biological or extended families acting as caregivers.
- Integrate prevention into humanitarian programming: All major aid programs must adopt a holistic, family-centric approach that addresses the needs of the entire family or caregiving unit, not just the separated child. This includes providing aid, counseling, and reintegration support to parents and community members to buffer against stigma and prevent the secondary separation that occurs due to economic strain or social rejection.
- Digitize and secure identity documentation: The international community should invest in technologies and standardized, secure, and easily verifiable digital civil registration systems (such as blockchain-based identity solutions) that are resilient to the chaos of mass displacement and destruction. This ensures that lost physical documents are not a permanent barrier to establishing identity and family lineage, which is critical for FTR.

## Footnotes

^1^Exclusion of non-English sources is noted as a limitation necessitated by limited time and resources.

## References

[ref1] Abdul Waajid M. Shrivastava G. (2017). Problems of conflict victimized orphans and community concerns in Kashmir. Int. J. Res. Available online at: https://journals.pen2print.org/index.php/ijr/article/download/11353/10806

[ref2] Alexander J. Boothby N. Wessells M. (2010). “Protection of children and youth affected by armed conflict: an essential link” in Protecting education from attack: a state-of-the-art review (Paris: UNESCO).

[ref3] American Academy of Child and Adolescent Psychiatry. (2023). Separating immigrant children from their families. Available online at: https://www.aacap.org/aacap/Policy_Statements/2018/Separating_Immigrant_Children_From_Their_Families.aspx

[ref4] American Immigration Council. (2025). The impact of family separation on children. Available online at: https://www.americanimmigrationcouncil.org/blog/the-impact-of-family-separation-on-children/

[ref5] Arksey H. O’Malley L. (2005). Scoping studies: towards a methodological framework. Int. J. Soc. Res. Methodol. 8, 19–32. doi: 10.1080/1364557032000119616

[ref6] Asokan A. (2021). Conflict agenda: integrating child protection issues and children’s voices in peace processes. J. Public Int. Aff. Available online at: https://jpia.princeton.edu/news/united-nations-children-and-armed-conflict-agenda-integrating-child-protection-issues-and

[ref7] Barnert E. Katsanis S. H. Mishori R. Wagner J. K. Selden R. F. Madden D.. (2022). Using DNA to reunify separated migrant families. Science 327, 1154–1156. doi: 10.1126/science.abh3979 PMC9185756 34045324

[ref8] Betancourt T. Borisova I. I. Williams T. P. Brennan R. T. Whitfield T. H. de la Soudiere M.. (2010). Sierra Leone’s former child soldiers: a follow-up study of psychosocial adjustment and community reintegration. Child Dev. 81, 1077–1095. doi: 10.1111/j.1467-8624.2010.01455.x, PMID: 20636683 PMC2921972

[ref9] Better Care Network. (2018). Children affected by armed conflict and displacement. Available online at: https://bettercarenetwork.org/library/particular-threats-to-childrens-care-and-protection/children-affected-by-armed-conflict-and-displacement

[ref10] Boothby N. (2008). Political violence and development: an ecological approach to children in war zones. Child Adolesc. Psychiatr. Clin. N. Am. 17, 497–514. doi: 10.1016/j.chc.2008.02.004 18558309

[ref11] Boothby N. Balster R. L. Goldman P. Wessells M. G. Zeanah C. H. Huebner G.. (2012). Coordinated and evidence-based policy and practice for protecting children outside of family care. Child Abuse Negl. 36, 743–751. doi: 10.1016/j.chiabu.2012.09.007, PMID: 23107455

[ref12] Brown B. (2000). Humanitarian intervention at a crossroads. William Mary Law Rev. 41:1683.

[ref13] Brown F. L. de Graaff A. M. Annan J. Betancourt T. S. (2017). Annual research review: breaking cycles of violence—a systematic review and common practice elements analysis of psychosocial interventions for children and youth affected by armed conflict. J. Child Psychol. Psychiatry 58, 507–524. doi: 10.1111/jcpp.12671, PMID: 27943284

[ref14] Bunn M. Samuels G. Higson-Smith C. (2023). Ambiguous loss of home: Syrian refugees and the process of losing and remaking home. Wellb. Space Soc. 4:100136. doi: 10.1016/j.wss.2023.100136, PMID: 37476200 PMC10358717

[ref15] Burlingham D. Freud A. (1944). Infants without families. Crows Nest, NSW: Allen & Unwin.

[ref16] Chech P. (2017). A right to family reunification for persons granted international protection? The Strasbourg case-law, state sovereignty and EU harmonization, EU Immigration and Asylum Law and Policy. Available online at: https://eumigrationlawblog.eu/a-right-to-family-reunification-for-persons-under-international-protection-the-strasbourg-case-law-state-sovereignty-and-eu-harmonisation-2/?print=print

[ref17] Civilians in Conflict. (2023). Displacement in armed conflict and the protection of civilians. Available online at: https://civiliansinconflict.org/wp-content/uploads/2023/09/CIVIC_Displacement_Brief_Draft2.pdf

[ref18] Council of Europe. (2025). Council of Europe joins statement by the International Coalition for the Return of Ukrainian Children. Available online at: https://www.coe.int/en/web/portal/-/council-of-europe-joins-call-on-russia-to-immediately-return-unlawfully-transferred-and-deported-children

[ref19] Elkhaili S. Sempijja N. (2025). The role of education in reintegrating ex-child soldiers: the case of Sierra Leone. Cogent. Educ. 12:2466307. doi: 10.1080/2331186X.2025.2466307

[ref20] Erlewein K. Gossmann E. Fegert J. M. (2024). Parental conscription and cumulative adverse experiences in war-affected children and adolescents and their impact on mental: a comment following Russia’s invasion of Ukraine, 2022. Child Adolesc. Psychiatry Ment. Health 18:42. doi: 10.1186/s13034-024-00732-0 38553764 PMC10981359

[ref21] European Council. (2025). Statement by International Coalition for the Return of Ukrainian Children of the Unlawful Deportation and Forced Transfer of Ukrainian Children by the Russian Federation (August 2025). Available online at: https://www.diplomatie.gouv.fr/en/country-files/ukraine/news/article/statement-by-the-international-coalition-for-the-return-of-ukrainian-children

[ref22] Feinstein International Center. (2017). The impact of protection interventions on unaccompanied and separated children: a systematic review. Available online at: https://fic.tufts.edu/wp-content/uploads/Child-Protection-Systematic-Review.pdf

[ref23] Ferguson S. (2018). Family separations: the hidden casualties of South Sudan’s civil war. Available online at: https://www.unicefusa.org/stories/family-separations-hidden-casualties-south-sudans-civil-war

[ref24] Georgetown University Collaborative on Global Children’s Issues. (2023). International law is unambiguous when it comes to protecting children in the midst of conflict. Available online at: https://globalchildren.georgetown.edu/posts/international-law-is-unambiguous-when-it-comes-to-protecting-children-in-the-midst-of-conflict

[ref25] Global Action on Aging. (n.d.) Work of intergovernmental and nongovernmental organizations. Available online at: https://globalag.igc.org/armedconflict/workofngos.htm

[ref26] Global Protection Cluster. (n.d.) Child and forced family separation. Available online at: https://globalprotectioncluster.org/Child_and_Forced_Family_Separation

[ref27] Gulati J. Khoso I. (2013). Re-envisaging the international law of internal armed conflict. Denver J. Int. Law Policy 41, 397–416.

[ref28] Habbach H. (2020). “You will never see your child again”: the persistent psychological effects of family separation. Available online at: https://phr.org/our-work/resources/you-will-never-see-your-child-again-the-persistent-psychological-effects-of-family-separation/

[ref29] Hague Conference on Private International Law. (2020). Information note—children deprived of their family environment due to the armed conflict in Ukraine. Available online at: https://www.hcch.net/en/news-archive/details/?varevent=854

[ref30] HDPI. (n.d.) Affiliates. Available online at: https://hdpi.org/who-we-are/affiliates/

[ref31] Hope and Home for Children. (n.d.) The harm of orphanages (part 2): weakening family and community structures in Africa. Available online at: https://www.hopeandhomes.org/blog/the-harm-of-orphanages-part-2-weakening-family-and-community-structures-in-africa/

[ref32] Human Rights Watch. (2000). Sierra Leone rebels forcefully recruit child soldiers. Available online at: https://www.hrw.org/news/2000/05/31/sierra-leone-rebels-forcefully-recruit-child-soldiers

[ref33] Human Rights Watch. (2003). Lasting wound: consequences of genocide and war for Rwanda’s children. Available online at: https://www.hrw.org/reports/2003/rwanda0403/rwanda0403.pdf

[ref34] Human Rights Watch. (2020). Deadly consequences: obstruction of aid in Yemen during COVID-19. Available online at: https://www.hrw.org/report/2020/09/14/deadly-consequences/obstruction-aid-yemen-during-covid-19

[ref35] Human Rights Watch. (2023). We must provide a family, not rebuild orphanages: the consequences of Russia’s invasion of Ukraine for children in Ukrainian residential institutions. Available online at: https://www.hrw.org/report/2023/03/13/we-must-provide-family-not-rebuild-orphanages/consequences-russias-invasion

[ref36] Human Rights Watch, Texas Civil Rights Project, & Yale Law School's Lowenstein International Human Rights Clinic. (2024). “We Need to Take Away Children”: zero accountability six years after “Zero Tolerance”. Available online at: https://www.hrw.org/report/2024/12/16/we-need-take-away-children/zero-accountability-six-years-after-zero-tolerance

[ref37] Ibhawph B. (2020). Refugees, evacuees, and repatriates: Biafran children, UNHCR, and the politics of international humanitarianism in the Nigerian Civil War. Afr. Stud. Rev. 63, 568–592. doi: 10.1017/asr.2020.43

[ref38] Interaction. (2025). Opportunities and barriers to preventing the recruitment and use of children by armed forces and armed groups. Available online at: https://protection.interaction.org/case-examples/opportunities-and-barriers-to-preventing-the-recruitment-and-use-of-children-by-armed-forces-and-armed-groups/

[ref39] Internal Displacement Monitoring Centre. (n.d.). Available online at: https://www.internal-displacement.org/internal-displacement/

[ref40] International Commission on Missing Person. (2023a). Available online at: https://icmp.int/2023-annual-report-highlights/

[ref41] International Commission on Missing Person. (2023b). Bosnia and Herzegovina. Available online at: https://icmp.int/the-missing/where-are-the-missing/bosnia-and-herzegovina/

[ref42] International Committee of the Red Cross. (2004). Inter-agency guiding principles on unaccompanied and separated children. Available online at: https://www.icrc.org/sites/default/files/external/doc/en/assets/files/other/icrc_002_1011.pdf

[ref43] International Committee of the Red Cross. (2013). Children associated with armed forces or armed groups. Available online at: https://www.icrc.org/sites/default/files/external/doc/en/assets/files/publications/icrc-002-0824.pdf

[ref44] International Committee of the Red Cross. (n.d.1). Customary IHL—Rule 105. Respect for family life. Available online at: https://ihl-databases.icrc.org/en/customary-ihl/v1/rule105

[ref45] International Committee of the Red Cross. (n.d.2). Customary IHL—Rule 135. Children. Available online at: https://ihl-databases.icrc.org/en/customary-ihl/v1/rule135

[ref46] International Committee of the Red Cross. (n.d.3). Customary IHL—Rule 131. Treatment of displaced persons. Available online at: https://ihl-databases.icrc.org/en/customary-ihl/v1/rule131

[ref47] International Committee of the Red Cross. (n.d.4). IHL Treaties—Additional Protocol (I) to the Geneva Conventions, 1977. Available online at: https://ihl-databases.icrc.org/en/ihl-treaties/api-1977/article-74/commentary/1987?activeTab=

[ref48] International Committee of the Red Cross. (n.d.5). Protected persons: children. Available online at: https://www.icrc.org/en/law-and-policy/protected-persons-children

[ref49] International Committee of the Red Cross. (n.d.6). Reconnecting families: preventing separation, searching for the missing, reuniting loved ones. Available online at: https://www.icrc.org/en/what-we-do/reconnecting-families

[ref50] International Organization for Migration. (2021). Global compact thematic paper: family reunification. Available online at: https://germany.iom.int/resources/global-compact-thematic-paper-family-reunification

[ref51] International Rescue Committee. (2024a). IRC reacts to latest IPC alert: famine is imminent in parts of Gaza. Available online at: https://www.rescue.org/press-release/irc-reacts-latest-ipc-alert-famine-imminent-parts-gaza

[ref52] International Rescue Committee. (2024b). Gaza Humanitarian Access, Snapshot #7: (10 October–13 November, 2024). Available online at: https://www.rescue.org/report/gaza-humanitarian-access-snapshot-7-10-october-13-november-2024

[ref53] Jones R. Johnson C. Brown W. Popescu G. Pallister-Wilkins P. Mountz A.. (2017). Interventions on the state of sovereignty at the border. Polit. Geogr. 59, 1–10. doi: 10.1016/j.polgeo.2017.02.006

[ref54] Jones-Mason K. Behrens K. Y. Gribneau Bahm N. I. (2019). The psychobiological consequences of child separation at the border: lessons learned from research on attachment and emotional regulation. Attach Hum. Dev. 23, 1–36. doi: 10.1080/14616734.2019.1692879 31769354

[ref55] Kelly J. Branham L. Decker M. R. (2016). Abducted children and youth in Lord’s resistance Army in northeastern Democratic Republic of the Congo (DRC): mechanisms of indoctrination and control. Confl. Heal. 10:11. doi: 10.1186/s13031-016-0078-5, PMID: 27195019 PMC4870729

[ref56] McCauley U. (2024). Separated children in South Sudan. Forced Migr. Rev.. Available online at: https://www.fmreview.org/mccauley/

[ref57] McNatt Z. Boothby N. (2018). Impact of separation on refugee families. Available online at: https://www.unhcr.org/wp-content/uploads/sites/27/2018/06/CH_Impact-of-Separation-on-Refugee-Families.pdf

[ref58] Mentzelopoulou M. (2025). Russia’s war on Ukraine: forcibly displaced Ukrainian children (briefing PDF) European Parliament. Available online at: https://www.europarl.europa.eu/RegData/etudes/BRIE/2023/747093/EPRS_BRI(2023)747093_EN.pdf

[ref59] Mousin C. (2019). Rights disappear when US policy engages children as weapons of deterrence. AMA J. Ethics 21, E58–E66. doi: 10.1001/amajethics.2019.58, PMID: 30672420

[ref60] Nabuco M. (2020). Family reunification as a right or a strategy to limit migration?. Available online at: https://www.imiscoe.org/news-and-blog/phd-blog/1002-family-reunification-as-a-right-or-a-strategy-to-limit-migration#:~:text=Since%20States%20cannot%20explicitly%20deny,the%20right%20to%20family%20life

[ref61] Ödebrink C. (2025). Russian abductions and deportations of Ukrainian children. Available online at: https://www.oscepa.org/en/documents/ad-hoc-committees-and-working-groups/parliamentary-support-team-for-ukraine/5294-russian-abductions-and-deportations-of-ukrainian-children-by-carina-oedebrink-eng/file

[ref62] Office of the High Commissioner for Human Rights. (1966). International covenant on civil and political rights. Available online at: https://www.ohchr.org/en/instruments-mechanisms/instruments/international-covenant-civil-and-political-rights

[ref63] Office of the High Commissioner for Human Rights. (1989). Convention on the rights of the child. Available online at: https://www.ohchr.org/en/instruments-mechanisms/instruments/convention-rights-child

[ref64] Office of the High Commissioner for Human Rights. (2025). Ukraine: devastating impact of hostilities on children’s rights. Available online at: https://www.ohchr.org/en/documents/country-reports/impact-armed-conflict-and-occupation-childrens-rights-ukraine-24-february

[ref65] Office of the Special Representative of the Secretary-General for Children in Armed Conflict. (n.d.). The Lord’s resistance army and children. Available online at: https://childrenandarmedconflict.un.org/2012/06/the-lords-resistance-army-and-children/

[ref9001] OHCHR. (1989). Convention on the rights of the child. Available online at: https://www.ohchr.org/en/instruments-mechanisms/instruments/convention-rights-child

[ref66] Østby G. Siri Rustad A. (2024). Children affected by armed conflict, 1990–2023, Conflict trends. Oslo: PRIO.

[ref67] PBS News. (2020). Why family separation is growing into a ‘global crisis’. Available online at: https://www.pbs.org/newshour/health/why-family-separation-is-growing-into-a-global-crisis

[ref9002] Physicians for Human Rights. (2020). You Will Never See Your Child Again: The Persistent Psychological Effects of Family Separation. Available online at: https://phr.org/our-work/resources/you-will-never-see-your-child-again-the-persistent-psychological-effects-of-family-separation/ (Accessed May 05, 2025).

[ref68] Picard Kentz & Rowe LLP. (2024). State sovereignty, intervention, and international law. Available online at: https://pkrllp.com/news-insights/state-sovereignty-intervention-and-international-law

[ref69] Pollock D. Peters M. D. J. Khalil H. McInerney P. Alexander L. Tricco A. C.. (2023). Recommendations for extraction, analysis, and presentation of results in scoping reviews. JBI Evid. Synth. 21, 520–532. doi: 10.11124/JBIES-22-00123 36081365

[ref70] Professionals in Humanitarian Assistance and Protection. (2021). Family tracing and reunification. Available online at: https://phap.org/theme-family-tracing-and-reunification

[ref71] Red Cross EU Office. (2015). Bringing families together. Available online at: https://redcross.eu/projects/bringing-families-together#:~:text=After%202015%2C%20in%20response%20to,years%20before%20applying%20for%20family

[ref72] ReliefWeb. (2025). Understanding child separation in humanitarian crises: insights from multisector needs assessments (2021–2023). Available online at: https://reliefweb.int/report/world/understanding-child-separation-humanitarian-crises-insights-multisector-needs-assessments-2021-2023

[ref73] Ressler E. (1988) in Unaccompanied children in emergencies: care and protection in wars, natural disasters, and refugee movements. eds. Boothby N. Steinbock D. J. (Oxford: Oxford University Press).

[ref74] Sandbu-Ryena H. (2019). 6,000 children reunited with families after years of separation in South Sudan. Available online at: https://www.unicef.org/press-releases/6000-children-reunited-families-after-years-separation-south-sudan

[ref75] Save the Children. (2017). The Rohingya refugee crisis. Available online at: https://www.savethechildren.org/us/what-we-do/emergency-response/rohingya-crisis

[ref76] Save the Children. (2023). New figures: 468 million children live in conflict zones. Available online at: https://www.savethechildren.org.uk/news/media-centre/press-releases/new-figures-millions-of-children-live-in-conflict-zones

[ref77] Schomerus M. (2007). “The Lord’s resistance army in Sudan: a history and overview” in Small arms survey (Geneva: Graduate Institute of International Studies).

[ref78] Security Council. (2024). UN documents for children and armed conflict: Security Council resolutions. Available online at: https://www.securitycouncilreport.org/un_documents_type/security-council-resolutions/?ctype=Children%20and%20Armed%20Conflict&cbtype=children-and-armed-conflict

[ref79] Sharp B. (n.d.). Misguided intentions: operation babylift and the consequences of humanitarian action. Available online at: https://ushistoryscene.com/article/operation-babylift/

[ref80] SOS Children’s Villages (2025). Five devastating effects of family separation on children—and how we can stop it. Available online at: https://www.sos-usa.org/news/topics/strengthening-families/5-effects

[ref81] St. Petersburg-USA Orphanage Research Team (2009). The effects of early social-emotional and relationship experience on the development of young orphanage children. The St. Petersburg-USA Orphanage Research Team. Monogr. Soc. Res. Child Dev. 73, vii–viii. doi: 10.1111/j.1540-5834.2008.00483.x PMC2702123 19121007

[ref82] The Alliance for Child Protection in Humanitarian Action. (n.d.) Guidance note: primary prevention of family separation. Available online at: https://alliancecpha.org/en/about-our-alliance

[ref83] Tol W. Song S. Jordans M. (2013). Annual research review: resilience and mental health in children and adolescents living in areas of armed conflict—a systematic review of findings in low- and middle-income countries. J. Child Psychol Psychiatry 54, 445–460. doi: 10.1111/jcpp.12053, PMID: 23414226

[ref84] UN. (2017). UNICEF and partner agencies in South Sudan help reunite 5,000 children and families. Available online at: https://africarenewal.un.org/en/magazine/unicef-and-partner-agencies-south-sudan-help-reunite-5000-children-families

[ref85] UN. (2024). The question of Palestine. Available online at: https://www.un.org/unispal/document/a-year-of-tears-12-months-of-war-on-children-unicef-report/

[ref86] UN. (2025). OCHA: Hostilities in Gaza city drive further displacement. Available online at: https://www.un.org/unispal/document/ocha-hostilities-in-gaza-city-drive-further-displacement/

[ref87] UN. (n.d.). Universal declaration of human rights. Available online at: https://www.un.org/en/about-us/universal-declaration-of-human-rights

[ref88] UN Research Guides. (2025). Convention on the rights of the child (1989) 35th anniversary. Available online at: https://research.un.org/c.php?g=1331357&p=9804957

[ref89] UNHCR. (2017). Orphaned Rohingya children forced to grow up too fast. Available online at: https://www.unhcr.org/news/stories/orphaned-rohingya-children-forced-grow-too-fast

[ref9003] UNHCR. (2020). Global Report 2020. Available online at: https://www.unhcr.org/wp-content/uploads/sites/27/2022/06/UNHCR-global-report-2020.pdf

[ref90] UNHCR. (2021). Family reunification. Available online at: https://www.unhcr.org/what-we-do/build-better-futures/solutions/complementary-pathways/family-reunification

[ref91] UNHCR. (2025). From separation to safety: rebuilding childhood after Sudan’s war. Available online at: https://www.unhcr.org/eg/news/stories/separation-safety-rebuilding-childhood-after-sudan-s-war

[ref92] UNHCR. (n.d.). Guidance note 9: Humanitarian evacuations, handbook for the protection of internationally displaced persons. Available online at: https://www.unhcr.org/sites/default/files/legacy-pdf/4794a5512.pdf

[ref93] UNHCR and UNICEF. (1992). Evacuation of children from conflict areas: considerations and guidelines. Available online at: https://www.refworld.org/policy/legalguidance/ia/1992/en/61735

[ref94] UNICEF. (1989). Convention of the rights of the child. Available online at: https://www.unicef.org/child-rights-convention/convention-text

[ref95] UNICEF. (2020). Family separation during crisis. Available online at: https://www.unicef.org/protection/family-separation-during-crisis

[ref96] UNICEF. (2021). Children in alternative care. Available online at: https://www.unicef.org/protection/children-in-alternative-care

[ref97] UNICEF. (2022). Crisis within a crisis: Rohingya refugee children separated from their parents. Available online at: https://www.unicef.org/bangladesh/en/crisis-within-crisis-rohingya-refugee-children-separated-their-parents-fire

[ref98] UNICEF. (2024). Separated by conflict: UNICEF reunites families. Available online at: https://www.unicef.org.au/stories/conflict-separated-families-reunification

[ref99] UNICEF USA. (2025). UNICEF reunites children separated from their families in Gaza. Available online at: https://www.unicefusa.org/stories/unicef-reunites-children-separated-their-parents-gaza

[ref100] UNICEF USA. (n.d.). Children in war and conflict. Available online at: https://www.unicefusa.org/what-unicef-does/emergency-response/conflict

[ref9004] United Nations. (2020). International Migration 2020. Available online at: https://www.un.org/development/desa/pd/sites/www.un.org.development.desa.pd/files/undesa_pd_2020_international_migration_highlights.pdf

[ref101] United Nations Security Council. (2009). Resolution 1882 adopted by the Security Council at its 617th Meeting (S/INF/65)

[ref102] van Dore K. Nhep R. (2021). Providing protection or enabling exploitation? Orphanages and modern slavery in post-disaster contexts. J. Mod. Slavery 6, 46–61. doi: 10.22150/jms/STCB4140

[ref103] Waddington M. (2020). Hope and homes for children welcomes the new lancet group commission. Lancet 7:667. doi: 10.1016/S2215-0366(20)30297-2 PMC7351419 32659218

[ref104] War Child. (2024). More than 150,000 children separated from their parents by war violence. Available online at: https://www.warchild.net/news/over-150000-children-seperated-from-parents-by-war-violence/

[ref105] Wessells M. (2016). Children and armed conflict: introduction and overview. Peace Conflict J. Peace Psychol. 22, 198–207. doi: 10.1037/pac0000176

[ref106] Williams E. D. Crews J. D. (2023). From dust to dust: ethical and practical issues involved in the location, exhumation, and identification of bodies from mass graves. Croat. Med. J. 44, 251–258. 12808715

[ref107] Woods M. (2025). Non-intervention (non-interference in domestic affairs). The Princeton encyclopedia of self-determination. Available online at: https://pesd.princeton.edu/node/551

[ref108] Yale School of Medicine. (2025). Fact sheet: Russia’s kidnapping and re-education of Ukraine’s children. Available online at: https://medicine.yale.edu/news-article/fact-sheet-russias-kidnapping-and-re-education-of-ukraines-children/

[ref109] Zack-Williams A. (2001). Child soldiers in the civil war in Sierra Leone. Rev. Afr. Polit. Econ. 28, 73–82. doi: 10.1080/03056240108704504

